# Development of air pressure mirroring particle dispersion method for producing high-density tungsten medical radiation shielding film

**DOI:** 10.1038/s41598-020-79819-5

**Published:** 2021-01-12

**Authors:** Seon-Chil Kim

**Affiliations:** Department of Biomedical Engineering, School of Medicine, Keimyung University, 1095 Dalgubel-daero, Daegu, 42601 Korea

**Keywords:** Biomedical engineering, Radiography

## Abstract

Radiation shielding films used in medical institutions are manufactured by mixing polymer materials with eco-friendly shielding materials. However, it is not easy to distribute the shielding material particles uniformly during the process. The uniform distribution of the shielding material particles is key to the reproducibility of the shielding performance of the films. Therefore, in this study, an air pressure mirroring particle dispersion method was developed to maintain a uniform distribution of the shielding material by dispersing the shielding material on a curved reflector through an air nozzle. The particle distribution state, density, and shielding performance of the cross-section and surface of the shielding films developed using the single-sided dispersion, double-dispersion, and air pressure mirroring particle dispersion methods were evaluated. Compared to the conventional single-sided distribution method, the shielding film produced by the air pressure mirroring particle dispersion method increased the particle packing by 41.5%, density by 12.9%, shielding material content by 22.2%, and shielding performance by 21.4%. Thus, the proposed dispersion method enables better shielding performance through uniform dispersion of shielding material, which is the most important parameter in the manufacture of low-dose shielding films.

## Introduction

With the increasing use of radiation in medical institutions for diagnosis and treatment purposes, the potential radiation risks for associated medical staff are also increasing^1^. In particular, radiation workers, including radiologists, nurses, and isotope handlers, are classified as medical personnel with a high risk of exposure to radiation and are regularly monitored through radiation instrumentation^2,3^.

The energy of X-rays used in the diagnostics is in the range where the photoelectric effect and Compton scattering occur frequently. The photoelectric effect occurs more frequently as its energy of radiation is lower and the atomic number of the photoelectron is higher, and is absorbed after interaction with a substance. In addition to the energy of the incident X-rays, Compton scattering also affects the radiation dose received by radiation workers by changing the traveling direction of radiation and thereby increasing the radiation dose to areas that are not directly exposed to the radiation source^4,5^. Traditionally, radiation workers have actively defended themselves against direct radiation exposure, for example, by maintaining a certain distance or wearing radiation shielding clothing. However, scattering rays, which typically occur in spaces where medical radiation is used, are low-dose indirect radiation, which also increases the risk of exposure. It has been reported that the sources of the scattering rays are the aperture and leakage from the X-ray unit as well as the patient’s body and the examination table. The dose from spatial scattering rays is 1.37–0.05 mGy/min, corresponding to a low radiation dose^6^. Low-dose radiation, such as natural radiation, is typically less than 100 mSv^7^.

The existing radiation shields are mainly made of lead. However, they are heavy and uncomfortable for daily wear. Therefore, a considerable amount of research is being conducted on lightweight materials. In fields where an X-ray generator is used at a distance, a shield is required for protection against the scattered rays rather than direct rays. It is necessary to investigate methods for shielding body parts that are not the direct examination areas in addition to designing shielding suits to shield low radiation doses for medical personnel who assist at a certain distance, such as an angiography room.

The transmission energy of scattered rays is weaker than that of direct rays; therefore, lighter radiation shields can be used. Heat-treated molded sheets that were conventionally used for radiation shielding purposes, were made of powdered lead and rubber; however, the lead can be replaced by bismuth, tungsten, antimony, barium, etc. Recently, products using tungsten have proven to be effective^8,9^. Tungsten, which is used as a lead substitute material in the manufacture of lightweight radiation shielding sheets, has a density of 19.3 g/cm^3^ and exhibits excellent radiation shielding performance, heat resistance, and abrasion resistance^10^.

In this study, instead of using a mixture of basic materials and polymeric compounds, tungsten nanopowder was distributed directly on film to produce a shielding film. Although it is insufficient for direct radiation, it is expected to be effective in shielding from low-dose indirect radiation. For the adsorption of tungsten nanopowder onto the film at high densities, it is important to consider the size and dispersion method of the powder particles. When the shielding material (tungsten) is high-filled, the density of the radiation shield increases and its thickness decreases; however, it can still easily block low-dose radiation^11,12^. Moreover, owing to the low thickness of the film, it has excellent workability and flexibility, and is expected to expand the range of use of various radiation shielding applications.

The existing shielding material mixing process has the disadvantage of increasing the porosity between the powder particles and decreasing particle packing (PP); moreover, the shielding performance is not uniform owing to the uneven application of particles^13,14^. To compensate for these shortcomings, this study has developed an air pressure mirroring particle dispersion method that directly disperses tungsten nanopowder on a film. The proposed air pressure mirroring particle dispersion method distributes the tungsten nanoparticles and air at high pressure on the film placed on curved reflectors, through two nozzles, thereby evenly distributing the constantly-sized tungsten nanoparticles and increasing the density of the shielding material. In order to understand the difference in the process between the shielding film made by dispersing the tungsten nanoparticles in the cross-section of the film surface and that made by dispersing the tungsten nanoparticles in two forms on both sides, each shielding film was produced and the distribution of the tungsten nanoparticles was observed. In addition, high-density radiation shielding can be developed by evaluating shielding performance and presenting a powder-filled molding method for producing a shielding film with low dose film thickness. We would like to present the relationship between the shielding performance of the radiation shielding film developed through such a process and the air pressure mirroring particle dispersion method, and propose standardization of the processing technology.

## Results

**Figure 1:**
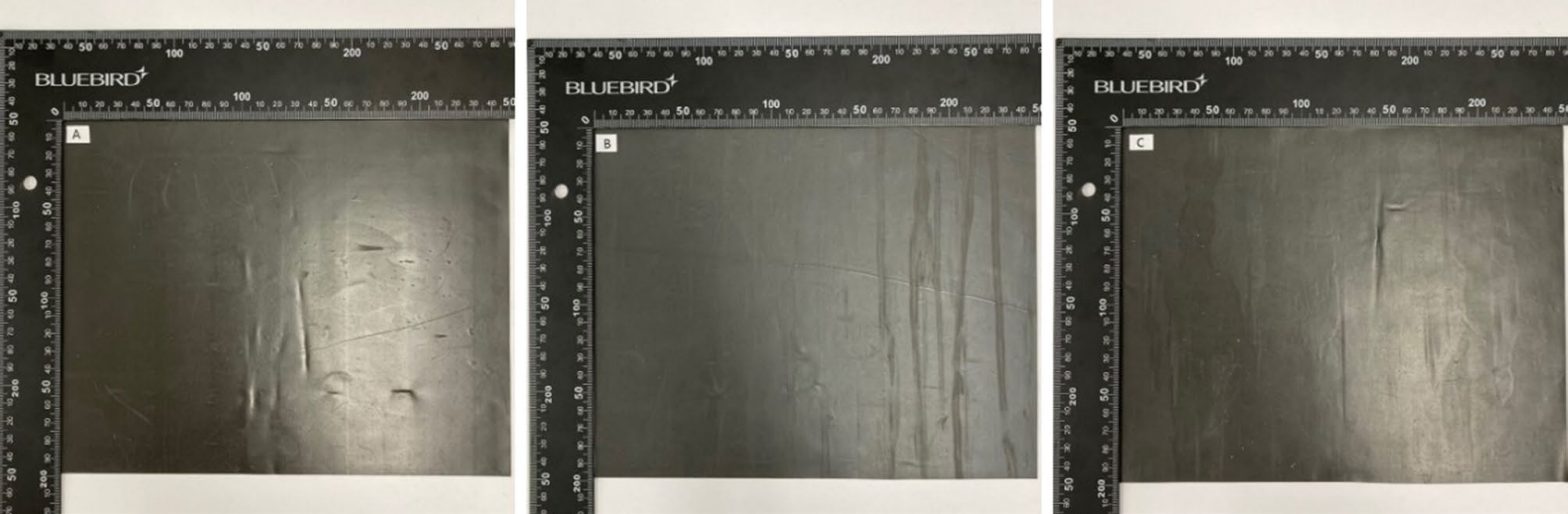
The three types of shielding films produced in the study. (**A**) Single-sided dispersion method shielding film (SSF, 25 cm × 20 cm). (**B**) Double-dispersion method shielding film (DSF, 25 cm × 20 cm), and (**C**) air pressure mirroring particle dispersion method shielding film (MSF, 25 cm × 20 cm).

The shielding films made using the tungsten nanopowder exhibit the characteristics presented in Table 1. Three shielding films manufactured by different shielding material dispersion methods were compared. All the shielding films were of the dimension 25 cm × 20 cm. A in Fig. 1 is a single-sided dispersion method shielding film (SSF), B, a double-dispersion method shielding film (DSF), and C, an air pressure mirroring particle dispersion method shielding film (MSF).

**Table 1:** Comparison of shielding films produced using the three different methods.

	Density (cm^3^)	Weight (g)	Thickness (mm)	Relative density	Tungsten particle packing (%)
Single-sided dispersion method shielding film (SSF)	6.277	113	0.45	0.54	56.5
Double-dispersion method shielding film (DSF)	6.615	156	0.65	0.61	78.0
Air pressure mirroring particle dispersion method shielding film (MSF)	7.090	172	0.55	0.62	80.0

As illustrated in Fig. 1, it is difficult to distinguish between the three types of shielding films by appearance. As can be seen in Table 1, the weights of the DSF and MSF were slightly more than that of the SSF, and the film thickness was the lowest for the MSF. When 200 g of tungsten nanopowder was dispersed in the same area, the SSF exhibited the largest loss. The MSF showed relatively good results with regard to density and relative density. Compared to the conventional SSF, the MSF had 41.5% higher PP, 12.9% higher density, and 22.2% higher shielding material content. When using the air pressure mirroring particle dispersion method, a standard processing technology that can adjust the thickness, density, and shielding performance according to the tungsten content can be applied. Therefore, as a result of this study, the shielding performance of the shielding film can be predicted by the amount of tungsten nanopowder. Through this, standard processing technology that can control the performance reproducibility of the shield to the amount of shielding material are possible.

**Figure 2:**
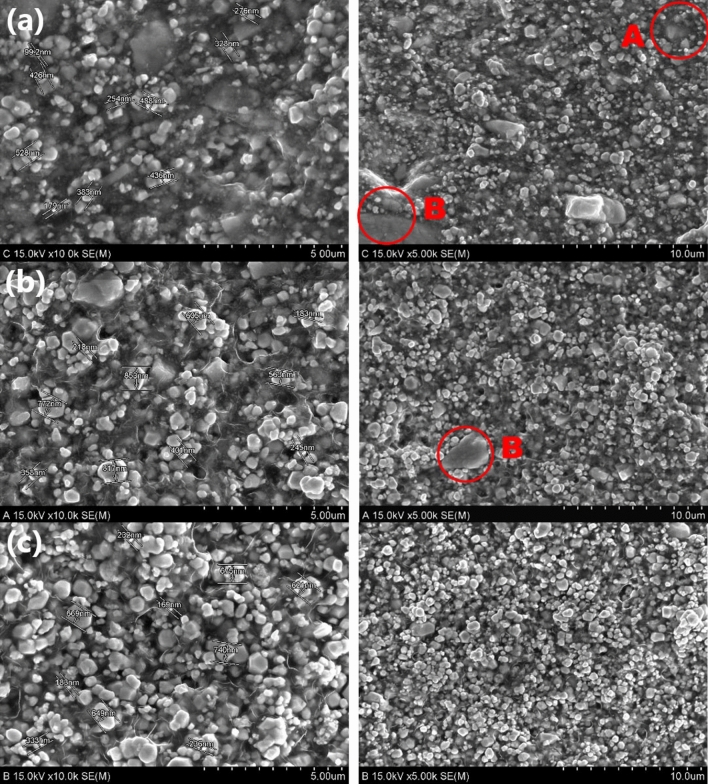
Comparison of images of tungsten particle distribution on shielding film cross-sections subjected to different dispersion methods. (**a**) Tungsten nanoparticles distribution on single-sided dispersion method shielding film (SSF). (**b**) Tungsten nanoparticles distribution on double-dispersion method shielding film (DSF). (**c**) Tungsten nanoparticles distribution on air pressure mirroring particle dispersion method shielding film (MSF).

**Figure 3:**
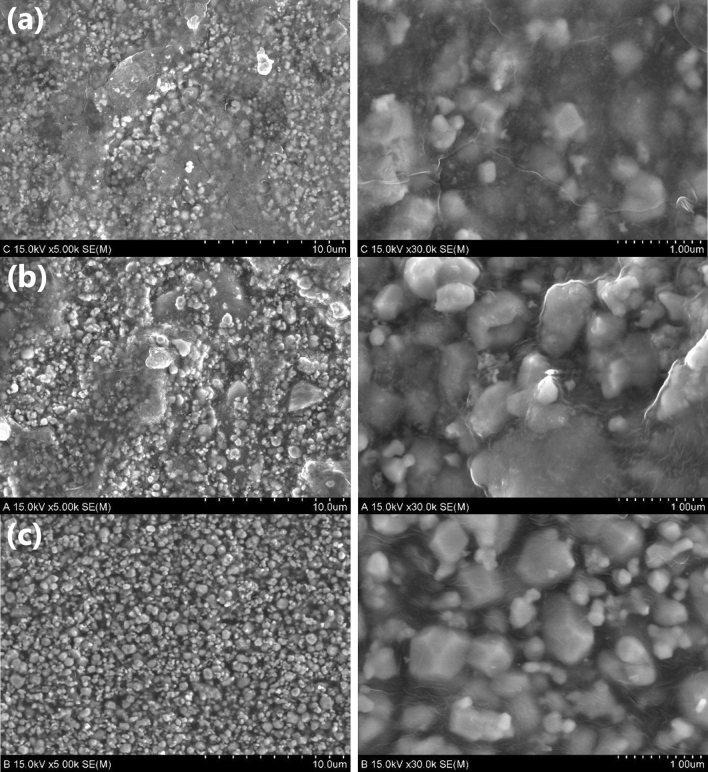
Comparison of images of tungsten particle distribution status on shielding film surface for different dispersion methods. (**a**) Shielding film surface that underwent single-sided dispersion method. (**b**) Shielding film surface that underwent double-dispersion method. (**c**) Shielding film surface that underwent air pressure mirroring particle dispersion method.

The distribution states of the tungsten nanoparticles in all three types of shielding films are depicted in Fig. 2. First, SSF and DSF were compared. In the double-dispersion method, the tungsten nanopowder was divided into two equal halves and double-distributed at the same location at high speeds to reduce voids and obtain a generally even distribution. In the air pressure mirroring particle dispersion method, where tungsten nanoparticles were dispersed twice in the same location on a curved reflector, the particles were generally evenly distributed. As depicted in Fig. 2a,b, the adhesive resins agglomerated and the particle distribution showed blank areas at places where the tungsten particles were not filled. A in Fig. 2a depicts a phenomenon in which some tungsten particles are lost during the last binding heat treatment process, and B in Fig. 2a shows the condensation of the adhesive resin on the polyethylene (PE) film. B in Fig. 2b can be found to have condensed adhesive resin on the PE film. The surface magnification images of the three shielding films show different distribution states of the tungsten nanoparticles, as depicted in Fig. 3. It can thus be confirmed that the air pressure mirroring particle dispersion method was the most effective in dispersing the tungsten nanoparticles on the surface.

**Figure 4:**
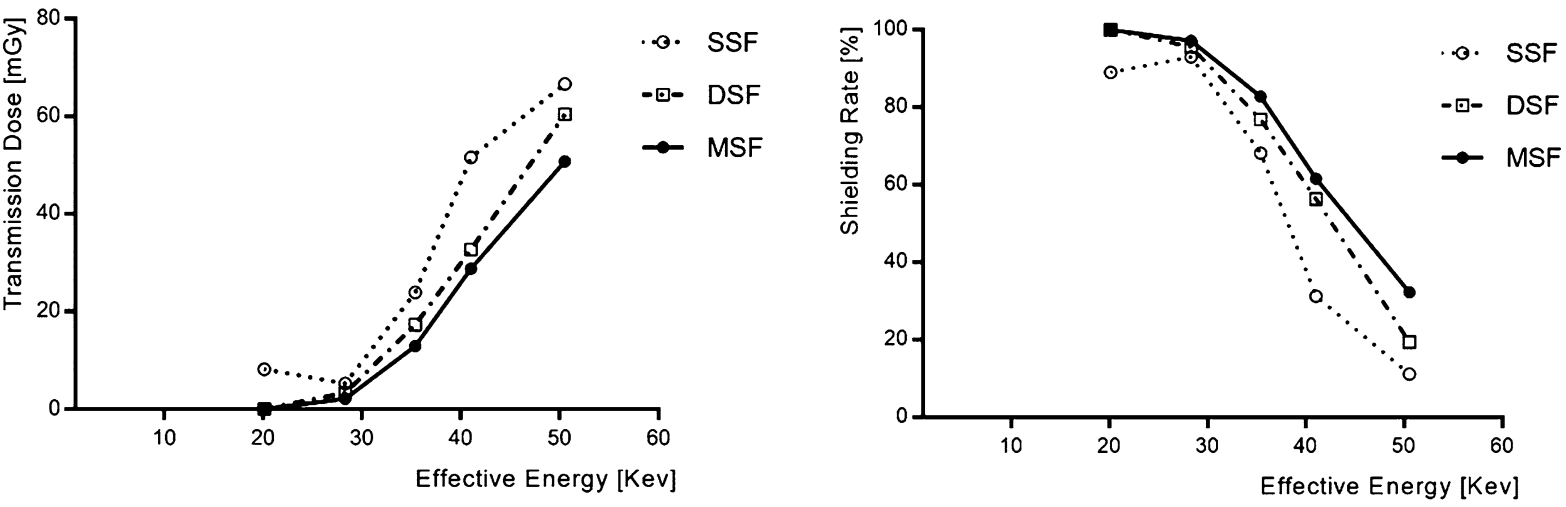
Comparison of transmission dose and shielding performance of Single-sided dispersion method Shielding film (SSF), Double-dispersion method Shielding film (DSF), and Air pressure mirroring particle dispersion method Shielding film (MSF). (**a**) Air pressure mirroring particle dispersion method with the curved reflector. (**b**) Single-sided dispersion method with plane reflector.

Next, the medical radiation shielding performances of the three types of films were evaluated. The X-ray effective energy used is presented in Table 2. The incident dose was 75 mGy, and the transmitted dose was the lowest in the MSF. Regarding the shielding performance, as can be seen in Table 3, in the low-energy region, both the DSF and the MSF appeared to be effective; however, there was a difference in the high-energy shielding performance, as shown in Fig. 4. In the SSF, the shielding performance was more effective at lower energy levels than that at higher energy levels. Overall, the MSF exhibited the best shielding performance. It can be observed that the three types of shielding films produced in this experiment showed different particle distributions of the shield material and also had different shielding performances.

**Table 2:** Comparison of effective energy to tube voltage.

Tube voltage (kV_p_)	Inherent filter (mmAl)	Added filter (mmCu)	Absorption coefficient (μ) (mm^−1^)	Half-value Layer (mmAl)	Effective energy (keV)
40	0.7	–	0.4665	1.15	20.14
60	0.7	–	0.2886	2.40	28.32
80	0.7	–	0.2015	3.15	35.41
100	0.7	0.2	0.1205	5.75	41.05
120	0.7	0.4	0.0912	6.87	50.54

**Table 3:** Transmitted dose and shielding rate according to shielding film.

Effective energy (keV)	A: Single-sided dispersion method shielding film (SSF)		B: Double-dispersion method shielding film (DSF)		C: Air pressure mirroring particle dispersion method shielding film (MSF)	
	Transmission dose (mGy)	Shielding rate (%)	Transmission dose (mGy)	Shielding rate (%)	Transmission dose (mGy)	Shielding rate (%)
20.14	8.2	89.07	0	100	0	100
28.32	5.2	93.07	3.275	95.63	2.119	97.17
35.41	23.87	68.17	17.32	76.91	12.91	82.79
41.05	51.55	31.27	32.71	56.39	28.77	61.64
50.54	66.62	11.17	60.41	19.45	50.76	32.32

## Discussion

Recently, in the medical field, with the development of preventive medicine, the number of tests using medical radiation has increased significantly; hence, exposure to medical radiation is also increasing^15^. There are three factors for radiation defense: time, distance, and shielding, but active shielding is the best way for protecting healthcare professionals working with patients^16^. From the patient's perspective, radiation exposure cannot be completely avoided for diagnostic and therapeutic purposes; however, one desires to have active protection with minimal exposure.

X-rays are absorbed by substances via the photoelectric effect, Compton scattering, electron pair generation, etc., during interaction with the human body, and the ionization makes shielding difficult in comparison with charged-particle radiation^17^. Moreover, the dose distribution varies depending on many variables, such as the energy level of radiation, the structure of the imaging room, the area of the examination room, and the imaging site^18^. Therefore, exposure to medical radiation, which corresponds to low doses, can easily occur and can be dealt with thin shields.

Thin shields are mostly made of bismuth, tungsten, antimony, barium sulfate, etc., using synthetic rubber and resin, among others, and a method for reducing the radiation pinholes by uniformly dispersing the shielding materials or increasing the density of shielding has been proposed^19^. The most common type of shield is a sheet made of synthetic resin mixed with metal powder. Another method involves making a sheet with different shielding materials to form a composite and then reconstructing the sheet into multiple layers.

**Figure 5:**
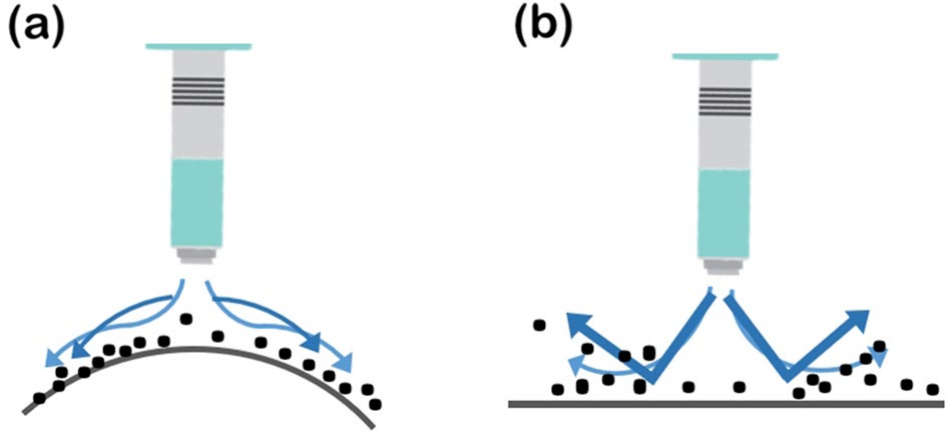
Principle of air pressure mirroring particle dispersion method and single-sided dispersion method.

The most important process parameter during the production of radiation shields is the density of the shield material, which has a direct relationship with the shielding performance. In this study, we proposed a processing technology for dispersing tungsten nanopowder on a PE film to satisfy the reproducibility of the shielding performance, and thereby tried to maintain the same density. The air pressure mirroring particle dispersion method was proposed to disperse the tungsten particles as evenly as possible on the PE film during dispensing. As depicted in Fig. 5b, the single-sided dispersion method shows poor PP because the tungsten particles get deflected due to the airflow. In contrast, as depicted in Fig. 5a the air pressure mirroring particle dispersion method uses a curved reflector, which causes the tungsten particles to adapt to the airflow, distribute evenly, and thereby increase PP. To reduce the thickness of the shielding film and maintain the thinness, increasing the PP per unit area and thereby increasing the density is the best method.

Dispersion of the shielding material is a key issue in the process of manufacturing the shielding film by mixing the shielding material and synthetic resin. For this purpose, various process techniques are used, such as mixing ratios and steps with additives^20^. The development of the processing technology of the radiation shield is related to the reproducibility of the uniform shielding performance in addition to the improvement of the shielding performance. Reproducibility and uniformity of the shielding performance are at the core of the radiation shielding film manufacturing technology. Even with the same shielding material and base material, there will be a difference in shielding performance depending on the manufacturing process. The shielding performance is affected by the composition of the shielding material and the polymer material and the degree of the additive reaction. This is because an even arrangement of particles reduces air gaps and can maintain the reproducibility of shielding performance. The dispersion method developed in this study is not a method of mixing a shielding material and a polymer, but a method of depositing and fixing the shielding material on a film. Therefore, the thickness of the shielding film can be adjusted, and the shielding performance can be predicted based on the content of the shielding material. The air pressure mirroring particle dispersion method makes it possible to realize a standard processing technology in which the shielding performance can be determined by the total amount of the shielding substance and the thickness of the shielding film. The shielding performance of currently used films is expressed by its lead equivalent, and it is difficult to predict the shielding performance from thickness. The method proposed in this study is expected to be able to solve this limitation.

The air pressure mirroring particle dispersion method showed satisfactory results for the improvement of shielding performance and reproducibility of uniform shielding performance.

## Conclusions

The air pressure mirroring particle dispersion method was proposed in this study for maintaining the reproducibility of shielding performance of eco-friendly radiation shielding films used in medical institutions improved the particle packing and uniformity of shielding materials. In this study, the shielding film, produced by applying the air pressure mirroring particle dispersion method, was compared with the shielding films produced by applying the double-dispersion and single-sided dispersion methods. The shielding material PP, particle distribution, density, and shielding performance of the shielding film produced by applying the air pressure mirroring particle dispersion method was superior to those of the shielding film produced by applying the single-sided dispersion method.

## Methods

The radiation intensity is affected by the distance and direction from the source generation point. The transmitted radiation intensity can be calculated using the thickness and density of the shielding film as follows^21^:

1 $$I={I}_{0}{\text{e}}^{-\upmu \text{x}}$$

$I$ Intensity of radiation that passes through the shield, ${I}_{0}$ Initial radiation intensity, $\upmu$ Linear attenuation coefficient, $\upchi$ Shield thickness (µm).

Radiation Intensity is attenuated through interaction with the shielding material as it passes through the material. Thus, by increasing the thickness or density of the medium, the radiation intensity can be lowered, and the shielding performance could be enhanced.

The absorption coefficient of the shielding film is directly related to the density and thickness of the shielding film containing the shielding material. This relation is derived as follows^22^:

2 $$\frac{\mu }{\rho }=\sum_{\text{i}}{w}_{\text{i}}{\left(\frac{\mu }{\rho }\right)}_{\text{i}}$$

$\frac{\mu }{\rho }$ Absorption coefficient, ${W}_{i}$ Mass ratio of tungsten.

As shown in Eq. (2), to improve the shielding performance, the radiation absorption coefficient must be increased along with the PP of the shielding material (tungsten nanopowder).

While manufacturing radiation shields, the density of the shielding film can be varied by controlling the thickness and area of the shielding film and by controlling the amount of dispersion on the shielding material. Thus, one way to decrease the thickness while maintaining shielding performance is to increase the PP of the shielding material at a constant volume^23^. The equation for obtaining the PP can be expressed as follows:

3 $$\text{PP}\begin{array}{c}={{\text{V}}_{\uprho }}/{{\text{V}}_{\text{f}}}=\left({{\text{W}}_{\text{m}}}/\text{V}\right)/{{\text{D}}_{\text{m}}}\end{array}$$

$\text{V}$: Volume of the shielding film, ${\text{V}}_{\uprho }$: Volume of the shield materials, ${\text{V}}_{\text{f}}$ : Volume of the shield base materials, ${\text{D}}_{\text{m}}$: Density of the shielding materials, ${\text{W}}_{\text{m}}$: Weight of the shielding materials.

The sizes of the tungsten nanoparticles manufactured externally (2020 BCTech co.Ltd) for this experiment are in the range of 120–480 nm. The reason why the same size is not used for all particles is because the particles are arranged freely according to their size to reduce air gap inside the shielding film. A PE film was used as the base material for the shielding film. When a radiation shielding sheet or a shielding film is produced using tungsten nanopowder, residual pores are generated. Radiation shields with residual pores show low fatigue resistance, poor radiation shielding performance, and limited flexibility. A PE film of thickness in the range 0.015–0.010 mm was used to compensate for these shortcomings^24^.

**Figure 6:**
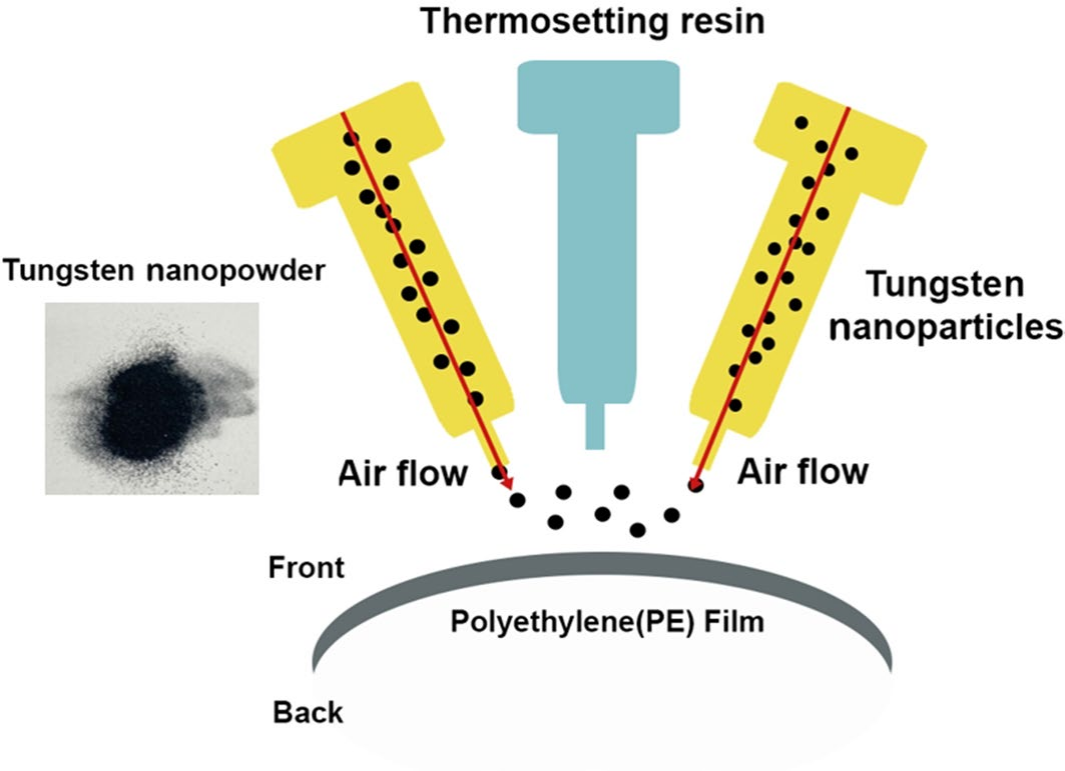
Fixing the dispersed particle on a PE film.

The air pressure dispersion method for uniformly dispersing tungsten nanoparticles in the PE film is illustrated in Fig. 6. The tungsten particles were directly dispersed onto the PE film through a thin air nozzle with a maximum diameter of 1.5 mm. However, in this method, tungsten nanoparticles get aggregated or scattered due to the turbulence generated at the nozzle inlet, leading to non-uniform dispersion of the nanoparticles. Therefore, the PE films were installed on curved reflectors and the air pressure mirroring particle dispersion method was employed. Mirroring refers to the dispensing of the same amount of a substance in the same location. Curved reflectors reduce the resistance at the nozzle openings during air pressure dispersion and thus help reduce the turbulence. In addition, a phenol resin, which is an adhesive of thermosetting resin, was dispersed in order to permanently fix the nanoparticles on the PE film. This was used to increase the affinity and PP of the tungsten nanopowder with the base material. A very small amount of less-than 5%/W phenol resin was added to 200 g tungsten nanopowder. The final tungsten shielding film then went through the binder process to correct its thickness, once again, at a constant pressure.

In this study, the tungsten shielding films used in the experiment for comparison were manufactured using three air pressure dispersion methods, and the influence of the dispersion state of the particles on the shielding performance was analyzed. The first shielding film was produced via the single-sided dispersion method, which also used an air nozzle for dispersion of the shielding material; however, the particles were dispersed by placing the PE film on a flat reflector. The second shielding film was produced via a double-dispersion method, in which tungsten nanopowder was divided in half and dispersed on a flat PE film twice. Finally, in the air pressure mirroring particle dispersion method, tungsten was dispersed through an air nozzle and the PE film was placed on a curved reflector.

The density of the tungsten nanopowder can be expressed as the relative density (RD) per unit area (m^2^) according to the particle distribution inside the shield, as shown below^25^:

4 $$RD=\frac{{e}_{\text{max}}-\text{e}}{{e}_{\text{max}}-{\text{e}}_{\text{min}}}=\frac{{1/D}_{\text{min}}-1/D}{{1/D}_{\text{min}}-{1/D}_{\text{max}}}$$

${e}_{max}$: Maximum gap ratio between particles, ${e}_{min}$: Minimum gap ratio between particles, ${D}_{max}$: Maximum density in unit area (g/cm^3^), ${D}_{min}$: Minimum density in unit area (g/cm^3^), $e$: Particle gap ratio in the shield, $D$: shielding film density, where the density of the shielding film can be presented in terms of particle spacing and shape.

The relative density is ideal for a result close to 1 inside the shield, and the amount of tungsten nanopowder can be calculated. Further, the relative density can be obtained from the volume of the shield per unit area. However, in the present study, the same shielding material amount was used. Further, a difference in the tungsten filling amount was observed owing to the dispersion process; the relative density was applied based on this difference.

It was also estimated that reducing the dispersion speed of the nozzle and narrowing the gap between the nozzles would reduce the air gap^26^. Field emissions scanning electron microscope (FESEM) was used to identify the particle distribution and the internal structure of the manufactured shielding films. Using FESEM, SEM image was analyzed under 15.0 kV × 10.0 k conditions.

Although the intensity of radiation used in the medical field is minimal, it has the ability to penetrate the human body. In this shield performance evaluation, the tube voltage, which accounts for the transmitted energy, was used as a reference, and the evaluation was performed using energy in five stages from 40 to 120 kVp. The experiment was conducted based on a tube current of 160 mA and an irradiation time of 60 ms. In addition, all X-ray energies used to obtain accurate transmitted energy were converted into effective energy and applied.

**Figure 7:**
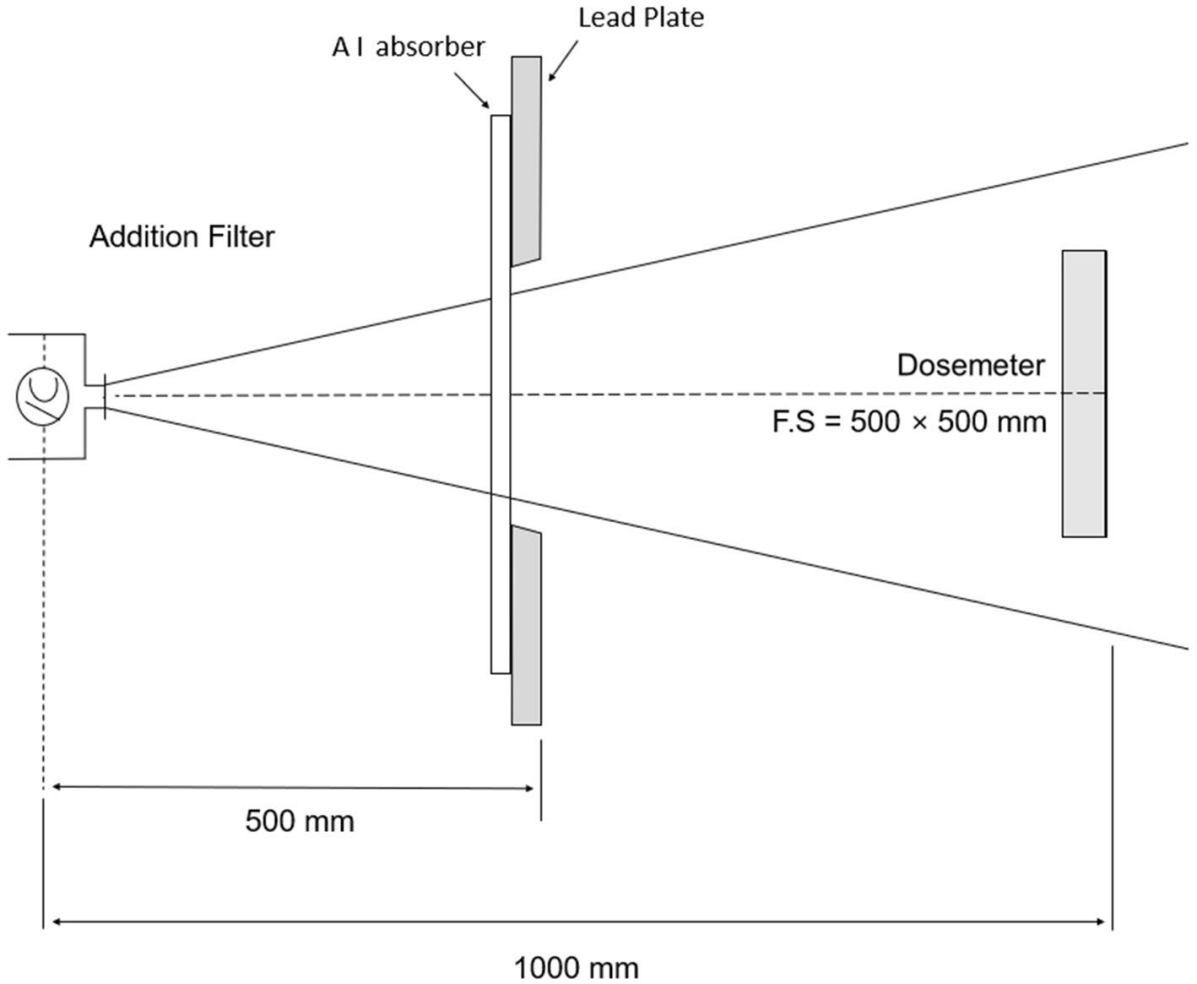
Measuring arrangement for half-value layer.

The geometric arrangement for measuring the effective energy of X-rays is shown in Fig. 7. The inherent filtration of the X-ray tube was fixed to 0.7 mmAl. The irradiation dose was measured while varying the thickness of the Al absorber in order to measure the half-value layer at each tube voltage. To measure the half-value layer, take $\text{log}e$ on both sides in Eq. (1); calculate the slope from the graph of $\text{y}=-{\upalpha \upchi }$; obtain the value of the linear absorption coefficient $\upmu$ from this slope. The half-value layer is then calculated as 0.693/$\upmu$. Hubbell’s mass absorption coefficient table is used to calculate the energy having a half-value layer corresponding to single energy^27^.

**Figure 8:**
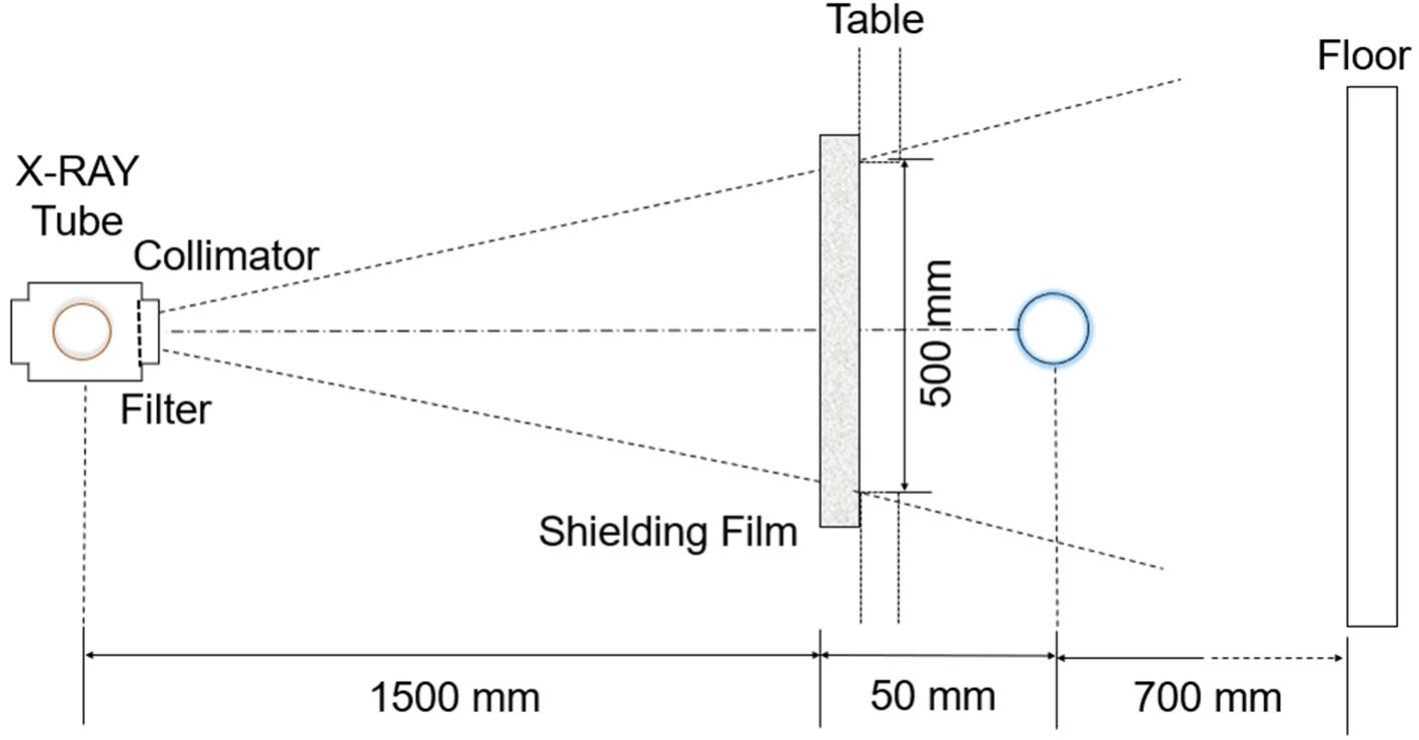
X-ray experiment to analyze the shielding performance of the shielding films.

The experimental setup to inspect the shielding performance of the three shielding films is shown in Fig. 8. The shielding rate of the shielding film was calculated as (1 − W/W0) × 100^28^. In this equation, W is the dose measured when there is a shielding film between the X-ray tube and the Dosemeter, and W0 is the irradiation dose value measured when there is no shielding film between the X-ray tube and the Dosemeter. A total of 10 experiments were performed using an X-ray generator (Toshiba E7239, 150 kV–500 mA, 1999, Japan) to obtain the transmissive dose values and the average of these values, given in Table 3, was used. The dose detector was tested and calibrated using DoshiMax plus 1 (2019 iba Dosimetry.Corp).
